# Defective nanomaterials for electrocatalysis oxygen reduction reaction

**DOI:** 10.3389/fchem.2022.1023617

**Published:** 2022-10-17

**Authors:** Zhanxin Mao, Xianyi Tang, Xuguang An, Jinxia Jiang

**Affiliations:** ^1^ National Hydrogen Power Quality Supervision and Inspection, China Automotive Engineering Research Institute Co.,Ltd., Chongqing, China; ^2^ Interdisciplinary Materials Research Center, Institute for Advanced Study, Chengdu University, Chengdu, China; ^3^ Chongqing Medical and Pharmaceutical College, Chongqing, China

**Keywords:** oxygen reduction reaction, defects, Pt and Pt-based alloy, N-C, metallic oxide

## Abstract

The difficulties in O_2_ molecule adsorption/activation, the cleavage of the O–O bond, and the desorption of the reaction intermediates at the surface of the electrodes make the cathodic oxygen reduction reaction (ORR) for fuel cells show extremely sluggish kinetics. Thus, it is important to the exploitation of highly active and stable electrocatalysts for the ORR to promote the performance and commercialization of fuel cells. Many studies have found that the defects affect the electron and the geometrical structure of the catalyst. The catalytic performance is enhanced by constructing defects to optimize the adsorption energy of substrates or intermediates. Unfortunately, still many issues are poorly recognized, such as the effect of defects (types, contents, and locations) in catalytic performance is unclear, and the catalytic mechanism of defective nanomaterials is lacking. In this review, the defective electrocatalysts divided into noble and non-noble metals for the ORR are highlighted and summarized. With the assistance of experimental results and theoretical calculations, the structure–activity relationships between defect engineering and catalytic performance have been clarified. Finally, after a deeper understanding of defect engineering, a rational design for efficient and robust ORR catalysts for PEMFCs is further guided.

## 1 Introduction

Hydrogen, as a kind of environment-friendly and high mass-energy density energy source, has great potential to replace fossil energy against the energy crisis and environmental pollution. Proton exchange membrane fuel cells (PEMFCs) or alkaline fuel cells (AFCs) are critical devices for hydrogen energy utilization (Li J. C. et al., 2020; Abdin et al., 2020). They can efficiently convert chemical energy from normal hydrogen into electric power. The sluggish kinetics of ORR on the cathode side is the most prominent issue, resulting in lower power density. It is urgent to develop highly active and stable ORR electrocatalysts (Nie et al., 2015; Ji et al., 2020).

Traditionally, Pt or Pt-based alloy is the most excellent ORR catalyst discovered so far. The commercial Pt/C catalysts can meet the requirements of some application scenarios with ultra-high Pt loading in membrane electrodes. However, oxygen or hydroxide species are too strongly adsorbed on the surface of Pt nanoparticles, which makes it difficult for desorption and thus increases the overpotential of the ORR (Stamenkovic et al., 2007). Although the alloying strategies, optimizing geometric structures, and reducing nanoparticle sizes have made a great improvement to the ORR performance of noble metal ORR catalysts (Li et al., 2022a; Feng et al., 2022), it is necessary to develop more sophisticated methods of catalyst preparation to achieve high-power fuel cells with ultra-low Pt mass loading set by the U.S. Department of Energy in 2025 (0.44 A mg^−1^ at 0.9 V at an ultralow Pt loading of 0.125 mg cm^−2^ in fuel cells). Furthermore, limited resources and the high price of Pt make it necessary to develop low-cost and abundant resources of non-noble metal catalysts. The N-doped carbon materials (N–C) and transition metal–nitrogen–carbon (M–N–C, M = Fe, Co, and Ni) complexes show excellent oxygen reduction activity, which can be prepared just by pyrolysis of N-containing organic precursors without or with metal elements (Cao et al., 2016; Wang et al., 2017; Xie et al., 2020; Yan et al., 2022). The enhanced ORR performance is achieved by optimizing the pore structure to a high specific surface area or increasing the atomic ratio of N and mono-dispersed metal elements to improve the number of active sites. “Fenton reaction” and corrosion of carbon materials at high potential lead these catalysts to suffer poor durability in acidic media (Han et al., 2021; Yang et al., 2021).

In general, defects are ubiquitous in solid materials and can be divided into four categories, namely, point defects, line defects, plane defects, and volume defects according to the size of the defect. The tiny regions deviating from the ideal crystal structure affect the fluctuation of crystal material properties, such as electrical, optical, magnetic, and chemical activity. The artificial control of defect types, contents, and locations makes defect engineering a common method in designing advanced functional materials (Wang et al., 2016; Chen, et al., 2016; Feng et al., 2020). In catalytic science, the defects could destroy the periodicity structure of the crystal and redistribute the local electronic state of the catalyst. For instance, the step edge can reduce the d-band center of the Pt or Pt-based alloy, making the intermediate species easier to desorption and thus improving its ORR activity. Bunched Pt–Ni alloy nanocages with rich low-coordination step atoms possess higher ORR activity and stability (Tian et al., 2019). However, a lack of theoretical and systematic understanding of diversity and complex defect structures has been preventing defects engineering to be an accurate way to regulate catalytic activity and stability (Xie et al., 2019). Recently, the development of the fine structure characterization techniques and theoretical computational chemistry, such as X-ray absorption fine structure spectrum (XAFS), spherical aberration-corrected transmission electron microscope (STEM), and density functional theory (DFT), enable understanding the relationship between defect structure and catalytic performance in depth. The types, contents, and locations of the defects can be more intuitively described and observed by these advanced characterization techniques, and the electronic structure of defective catalysts can be approximately calculated by theoretical calculation (Li W. et al., 2020). This makes it possible to correlate the relationship between defect structure and the adsorption energy of the substrate and intermediate species. Even though many researchers applied the defects to improve catalytic performance from various aspects, the role of the defects in the catalytic activity and stability is still necessary to be deeply discussed to form a systematic and comprehensive understanding. In this review, we systematically summarize the noble and non-noble defective ORR electrocatalysts from the tuning of the electronic structure to the optimizing of the adsorption energy and reaction path. It is expected that the relationship between the defective ORR electrocatalysts and their activity and stability can be clarified to guide the rational design of desirable catalysts.

## 2 The structure–activity relationship between defects and electrocatalytic performance

### 2.1 Noble metal catalysts

**FIGURE 1:**
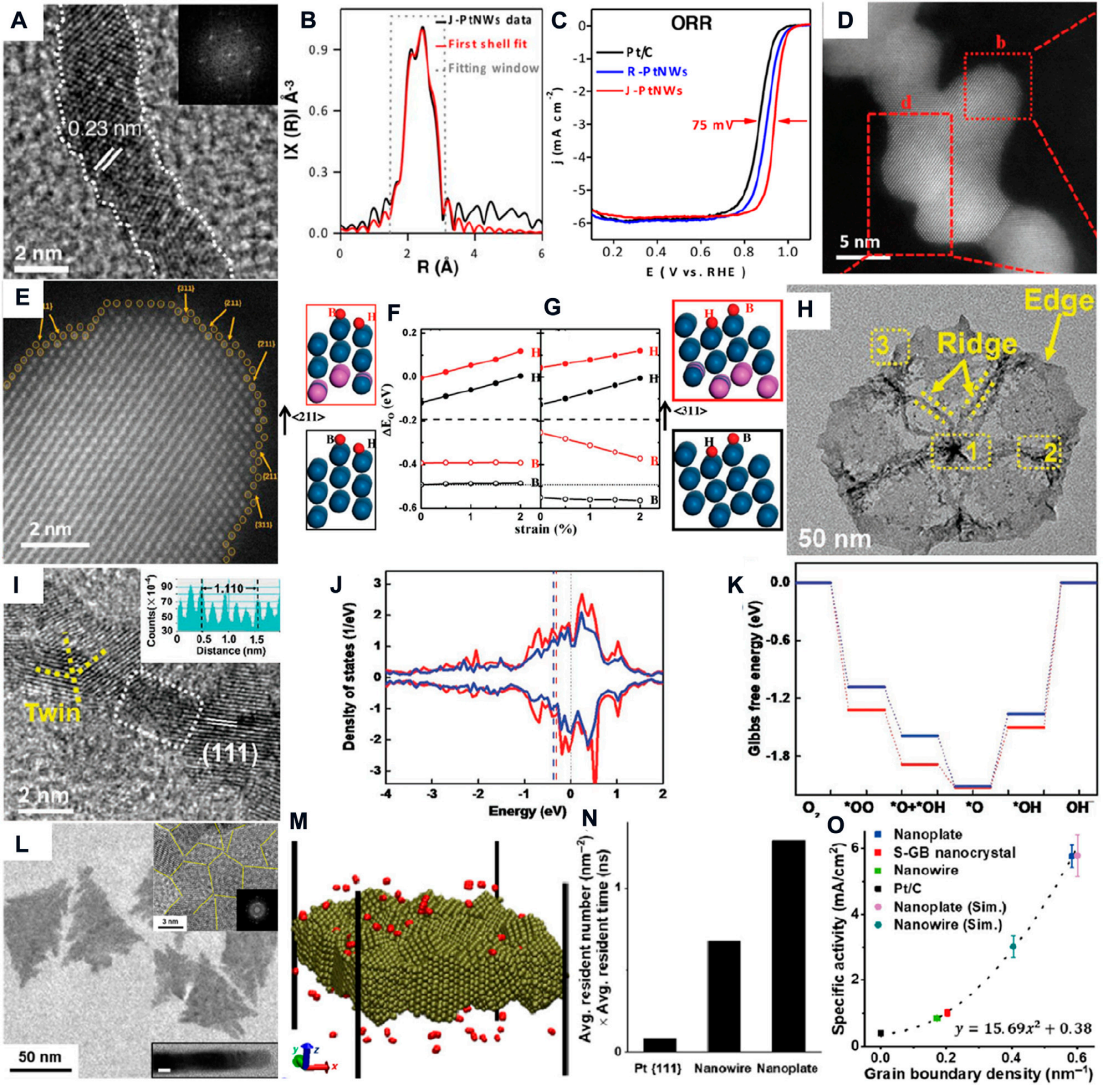
**(A)** HRTEM image and **(B)** Pt L_3_ edge FT-EXAFS spectrum of ultrafine jagged Pt nanowires; **(C)** LSV curves for the selected contrast catalyst (Li et al., 2016). **(D)** HAADF-STEM and **(E)** enlarged b red square images of Pt-skin zigzag-like Pt_3_Fe nanowires; DFT calculation of oxygen adsorption energy on **(F)** (211) and **(G)** (311) surfaces (Luo et al., 2018). **(H)** TEM and **(I)** HRTEM images of Pt–Cu–Mn ultrafine nanoframes; **(J)** bonding (Red) and antibonding (blue) states, respectively; **(K)** free energy of pristine (red) and compressed (blue) surfaces (Qin, et al., 2020). **(L)** TEM and HRTEM images of ultrathin Pt nanoplates; **(M)** Slab lattices of the Pt nanoplate; **(N)** product of the average oxygen resident number and time in different assembled nanostructures; **(O)** quantitative simulation curve between specific activities of the ORR and the grain boundary densities (Zhu et al., 2020).

**FIGURE 2:**
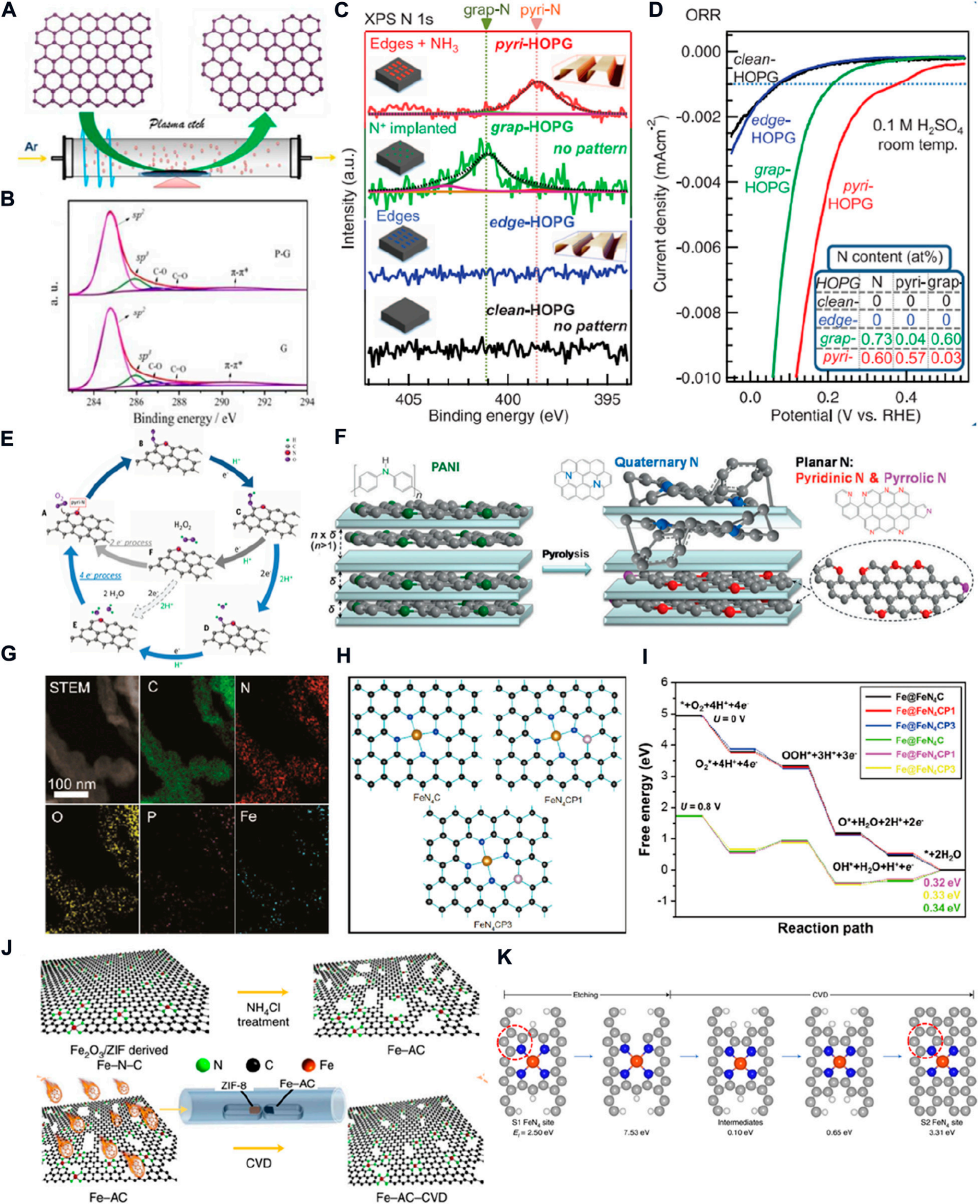
**(A)** Schematic diagram of the edge-rich graphene preparation. **(B)** C1s XPS spectra of graphene and plasma-treated graphene (Tao et al., 2016). **(C)** N1s XPS spectra of model catalysts; **(D)** LSV curves of model catalysts; **(E)** Schematic for the ORR pathway on N-doped carbon materials (Guo et al., 2016). **(F)** Schematic representation showing the selectivity inside and outside of montmorillonite during nitrogen-doped graphene synthesis (Ding et al., 2013). **(G)** STEM image and corresponding EDS element mapping of P-doped Fe-N-C; **(H)** models of FeN_4_CP1 and FeN_4_CP3; **(I)** free energy of the ORR on FeN_4_C, FeN_4_CP1, and FeN_4_CP3 sites (Li W. et al., 2020). **(J)** Schematic diagram of synthesizing a long-term durability Fe–N–C catalyst; **(K)** possible FeN_4_ site conversion during the CVD process calculated by DFT (Liu et al., 2022).

Over the past decades, noble metals are regarded as superior ORR electrocatalysts with a 4-electron pathway. Nørskov et al. present a volcanic diagram to describe the relationship between the active and O binding energy, which shows that Pt has the best ORR catalytic activity. However, the intermediate species (e.g., O^*^ and OH^*^) are strongly bonded on the surface of Pt catalysts, causing desorption of those species to be the crucial step (Nørskov et al., 2004; Stamenkovic et al., 2006). The step edges are the most widely studied for Pt ORR electrocatalysts. The step edges usually correspond to high index crystal faces, and the step atoms are coordinated unsaturated. The step atoms can optimize the electronic structure to lower the d-band center so that the intermediate species are easily desorbed from the surface to improve the ORR activity. For example, Duan’s group synthesized ultrafine jagged Pt nanowires with numerous step edges (Figure 1A). (Li et al., 2016) The FT-EXAFS spectrum shows that Pt–Pt long length is shorter than Pt foil, which indicates that the lattice of ultrafine jagged Pt nanowires is shirked compared to that of bulk Pt (Figure 2B). The coordination numbers of bulk Pt are 8 or 9 at (100) or (111) faces, respectively, while it is 6–8 in the ultrafine jagged Pt nanowires, and this is contributed by under-coordination step Pt atoms. As a result, the half-wave potential of ultrafine jagged Pt nanowires (0.935V vs. RHE) is higher than that of regular Pt nanowires (0.90 V vs. RHE) and commercial Pt/C (0.86 V vs. RHE), and the ORR performance has barely changed after 6000 cycles.

PtM alloy catalysts generally have better ORR activity than pure Pt catalysts due to more weakly bound oxygen species energy (about 0.2 eV) than bulk Pt. The role of 3d metals(M) as the solute element for Pt-based alloy electrocatalysts can cause the Pt lattice to shrink and change the surface electronic structure, lowering the d-band center. The ORR activity of PtM alloy can be further boosted by the introduction of surface defects (Bu et al., 2016; Li et al., 2022b). For instance, Guo et al. reported Pt-skin zigzag-like Pt_3_Fe nanowires, and the surfaces of Pt-skin zigzag-like Pt_3_Fe nanowires and zigzag-like Pt_3_Fe nanowires all exist with stable high-index facets and step atoms (Figure 1E). The DFT calculation of the bridge site (B) at the step edge and the hollow site (H) on the facet confirmed the oxygen adsorption energy of the high-index crystal faces with step atoms closer to the optimal value to superior ORR activity on the Pt-skin zigzag-like Pt_3_Fe nanowires (Figures 1F,G). The step atoms and the ligand effect cause the ORR activity of the Pt-skin zigzag-like Pt_3_Fe nanowires to be higher than that of most reported PtFe-based nanoparticle catalysts and zigzag-like Pt3Fe nanowires. The mass and specific activity are 2.1 and 1.9 times more than those of zigzag-like Pt^3^Fe nanowires, respectively. It only decreases 26.7% and 24.6% after 50000 cycles between 0.6 and 1.1 V versus RHE, respectively. (Luo et al., 2018).

Pt and Pt alloy twinned electrocatalysts possess numerous plane defects (stacking faults) at grain boundaries in twin crystals typically. Optimization of this kind of twin defects can tune the surface strain to make the surface electronic structure of the catalysis more suitable for oxygen species adsorption or desorption. (Zhang et al., 2017; Mariano et al., 2017; Chattot et al., 2018). For example, Qin et.al prepared penta-twinned ultrafine Pt–Cu–Mn nanoframes with high-density twin defects by a wet-chemical method (Figures 1H,I) (Qin, et al., 2020). It shows about 1.5% compressive strain caused by the increased proportion of twin defects than the Pt–Cu–Mn pentagonal nanoframes. The DFT calculation indicates that the compressive strain increases the occupation of anti-bonding states to lower the adsorption energy of OH^*^ on the compressed surface (Figures 1J–L). Thus, ultrafine Pt–Cu–Mn nanoframes emit the highest half-wave potential and mass activity of the ORR, which well-matches with the experimental results. The grain boundary densities may have a positive correlation to the ORR performance. Similarly, Yu Huang’s group compared ultrathin Pt nanoplates, Pt single-grain-boundary nanocrystals, Pt nanowires, and commercial Pt/C with different grain boundary densities to evaluate the ORR performance (Zhu et al., 2020). The results also suggest that the half-wave potential and mass activity have an order with the variation of grain boundary densities (ultrathin Pt nanoplates > Pt nanowires > Pt single-grain-boundary nanocrystals > commercial Pt/C), and the mass activity of ultrathin Pt nanoplates is 9.52, 10.7, and 13.72 times higher than that of Pt nanowires, Pt single-grain-boundary nanocrystals, and commercial Pt/C, respectively (Figure 1M). The strain is not observed in these assembled nanostructures, and the differences in ORR activity are attributed to the varied grain boundary density. The average resident number and time of oxygen residence on the surfaces are 15.02 times higher on ultrathin Pt nanoplates than on Pt single-grain-boundary nanocrystals by the molecular dynamic simulations (Figures 1N,O). Finally, a quantitative simulation curve to the quadratic equation ( $y=15.69x^{2}+0.38$ ) is established for the specific activities and the grain boundary densities (Figure 1P).

### 2.2 Non-noble metal catalysts

#### 2.2.1 Defective carbon-based catalysts

For wide application of PEMFCs, it is important to explore low-cost, high-activity, and durable catalysts to replace noble metal ORR catalysts. Carbon nanomaterials with excellent electrical conductivity and tolerance to acidic/alkaline media are facile to synthesize at low prices. The high surface area and controllable pore structure are conducive to carrying more active sites and material transfer. It has attracted considerable interest to replace noble metal ORR catalysts (Xu et al., 2020). However, the oxygen molecules are difficult to be adsorbed and activated on their surface. The modulation of spin distribution or charge transfer on the *sp* ^*2*^ carbon plane can greatly improve the ORR activity. As shown in Figures 2A,B, the C1s peaks of graphene and plasma-treated graphene show that the ratio of C–C (*sp* ^*2*^) to defect peak (*sp* ^*3*^) decreased from 8.88 to 6.35. It indicated that the intrinsic defects are constructed to form edge-rich graphene. The electronic structure of unsaturated *sp* ^*3*^ carbon atoms on the edge sites is more capable of oxygen adsorption and O–O bond cleavage than that of *sp* ^*2*^ C atoms. With the increase of the density for edge sites, the ORR activity is significantly improved. (Tao et al., 2016).

The limit of activity is determined by the intrinsic activity of active sites. Compared to intrinsic defects, the doped defects in carbon-based materials are more effective in improving intrinsic activity. The heteroatoms can regulate the charge density or spin distribution of neighboring carbon atoms (Zhang and Xia, 2011). The N element is considered to be the most effective among the heteroatoms that can be doped. The N-doped carbon materials can be prepared by a simple method, such as carbonizing N-containing organic polymers or annealing carbon materials under an NH_3_ atmosphere. The doped-N are present in four types: pyridinic-N, quaternary-N (graphitic-N), pyrrolic-N, and oxidized-N. (Yan et al., 2017). Owing to N species being randomly formed in the pyrolysis progress, a single structure of doped-N is hard to be prepared for understanding the role of different types of N species (Xia et al., 2016). Therefore, the different types of N-dominated model catalysts are prepared by mild bombardment with a nitrogen ion and Ar^+^ beam or annealing under the NH_3_ atmosphere of highly oriented pyrolytic graphite (HOPG) with well-defined π conjugation (Guo et al., 2016). The N1s XPS results confirm that the N concentrations of model catalysts are 0.73 atomic % of graphitic N-dominated HOPG and 0.60 atomic % of pyridinic N-dominated HOPG (Figure 2C). As shown in Figure 2D, the pyridinic N-dominated HOPG with low nitrogen content and a high ratio of pyridinic-N possesses the highest ORR activity than other model catalysts, and the ORR activity is positively correlated with the density of pyridinic-N. The acidic CO_2_ molecules are only adsorbed on the pyridinic N-dominated HOPG. This phenomenon and DFT calculation confirmed that the pyridinic-N reconstructs the electronic structure of neighboring C atoms to exist in a localized density of states in the occupied region near the Fermi level. Thus, the C atoms can donate electron pairs to behave as the Lewis base site to reduce O_2_. A possible four-electron or 2 + 2-electron ORR mechanism on this Lewis base site is proposed in Figure 2E. In detail, the four-electron mechanism occurs at a single site, and the protonated O_2_ molecules directly break to form OH species and then into two H_2_O molecules. For the 2 + 2-electron pathway, the protonated O_2_ molecules form H_2_O_2_ with another proton and are desorbed from the site. Then, the H_2_O_2_ is reabsorbed on another site to be reduced by two protons to generate H_2_O. However, the insufficient stability of pyridine-N at high temperatures results in the high density of pyridine-N electrocatalysts, making it very hard to prepare by pyrolysis. Most studies just attempted to increase the ratio of N elements, construct a porous structure of high specific surface area, or build an abundance of three-phase interfaces to improve ORR activity. The pyridinic-N and pyrrolic-N are in the same plane as the C atoms, the so-called plane-N. The graphitic-N would cause local distortion of the carbon lattice to a non-plane structure. Thus, Wei’s group developed a space-confinement-induced synthesis method to precisely regulate N species in pyrosis (Figure 2F). (Ding et al., 2013) The layered montmorillonite was selected as a quasi-closed flat nanoreactor for intercalation of aniline into the montmorillonite for self-assembly polymerization as a precursor of pyrolysis. The N/C ratio and the content of plane-N (especially pyridine-N) could be increased by optimizing the interspace width of montmorillonite. As a result, the catalysts, which were obtained at the interspace width of 0.46 nm, exhibited the highest content of N elements and pyridine-N, achieving the optimized ORR activity in an acidic medium. The half-wave potential lagged only 60 mV behind the commercial Pt/C with lower H_2_O_2_ production, which means that the ORR process on pyridine-N tends to be a four-electron pathway.

The M–N–C catalysts have better ORR catalytic activity than N–C and even exceed the Pt/C in an alkaline medium. The intrinsic activity of Fe–N–C was higher than that of Co/Ni/Cu–N–C and considered the most potential substitute for Pt-based catalysts. However, there is still a considerable gap in the intrinsic activity between the Fe–N–C- and Pt-based catalysts. Doping non-metallic elements in Fe–N–C catalysts can change the local electronic states of FeN_4_, further improving the ORR activity. As shown in Figure 2G, the phytic acid was added as a P source in the pyrolysis process to form P-doped Fe–N–C. The low formation energies of FeN_4_ CP1 and FeN_4_ CP3 models shown in Figure 2H were the most likely structure by DFT calculation. The localized state density of FeN_4_C sites could be optimized by the P bonded with the C atom around FeN_4_C to promote ORR activity. The free energy diagram shows that irrespective of applied potential of 0 V or 0.8 V, the FeN_4_CP1 and FeN_4_CP3 exists lower theoretical overpotential and thermodynamic overpotential than the FeN_4_C (Figure 2I). The experimental results also showed that the half-wave potential is increased from 0.835 to 0.858 V after doping P. (Li J. C. et al., 2020).

Although Fe–N–C has a relatively high ORR activity, poor stability is another key issue against its application. There are two types of active sites with different coordination environments for the ORR in Fe-N–C catalysts. One is the FeN_4_C_12_ site with four pyrrole-N as ligands to the Fe atom, and the other is the FeN_4_C_10_ site with four pyridine-N ligands. The FeN_4_C_12_ site shows better ORR intrinsic activity but poor stability to the FeN_4_C_10_ site. The FeN_4_C_12_ site is irreversibly oxidized to Fe_2_O_3_ in ORR progress, and the FeN_4_C_10_ site has stronger resistance to Fe atom stripping and oxidation (Kramm et al., 2012; Zitolo et al., 2015; Wan and Shui, 2022). Therefore, regulating the ratio of the two type sites in the Fe-N-C catalyst to balance the activity and stability is very important.

Recently, a long-term durable Fe-N-C catalyst was synthesized *via* chemical vapor deposition of a thin N–C on the surface of an NH_4_Cl-treated Fe–N–C catalyst (Figure 2J). The DFT calculation suggested that the formation energy of the FeN_4_C_12_ is more positive than that of the FeN_4_C10 and the kinetics is sluggish for the conversion of FeN_4_C12 to FeN_4_C10. The research found that the carbon defects constructed by NH_4_Cl treatment play a decisive role in the preparation of this catalyst. The highly active FeN_4_C12 site surrounded by rich carbon defects will be transformed into the high-stability FeN_4_C10 site in the chemical vapor deposition process. A possible evolutionary pathway is shown in Figure 2K by DFT calculation. The carbon defects reduce the active energy and accelerate the kinetics of this transformation. The vacant sites would introduce the transformation of pyrrolic-N into pyridinic-N in the chemical vapor deposition process. Finally, the Fe-N-C catalyst contains 65% FeN_4_C_12_ and 29% FeN_4_C_12_ to be a more stable catalyst with 53% FeN_4_C_12_ and 42% FeN_4_C_12_. This active site transformation balances catalytic activity and stability of the Fe–N–C catalyst, and a long-term stable cycle is achieved in H_2_/O_2_ fuel cells. (Liu et al., 2022).

#### 2.2.2 Metal oxide catalysts

**FIGURE 3:**
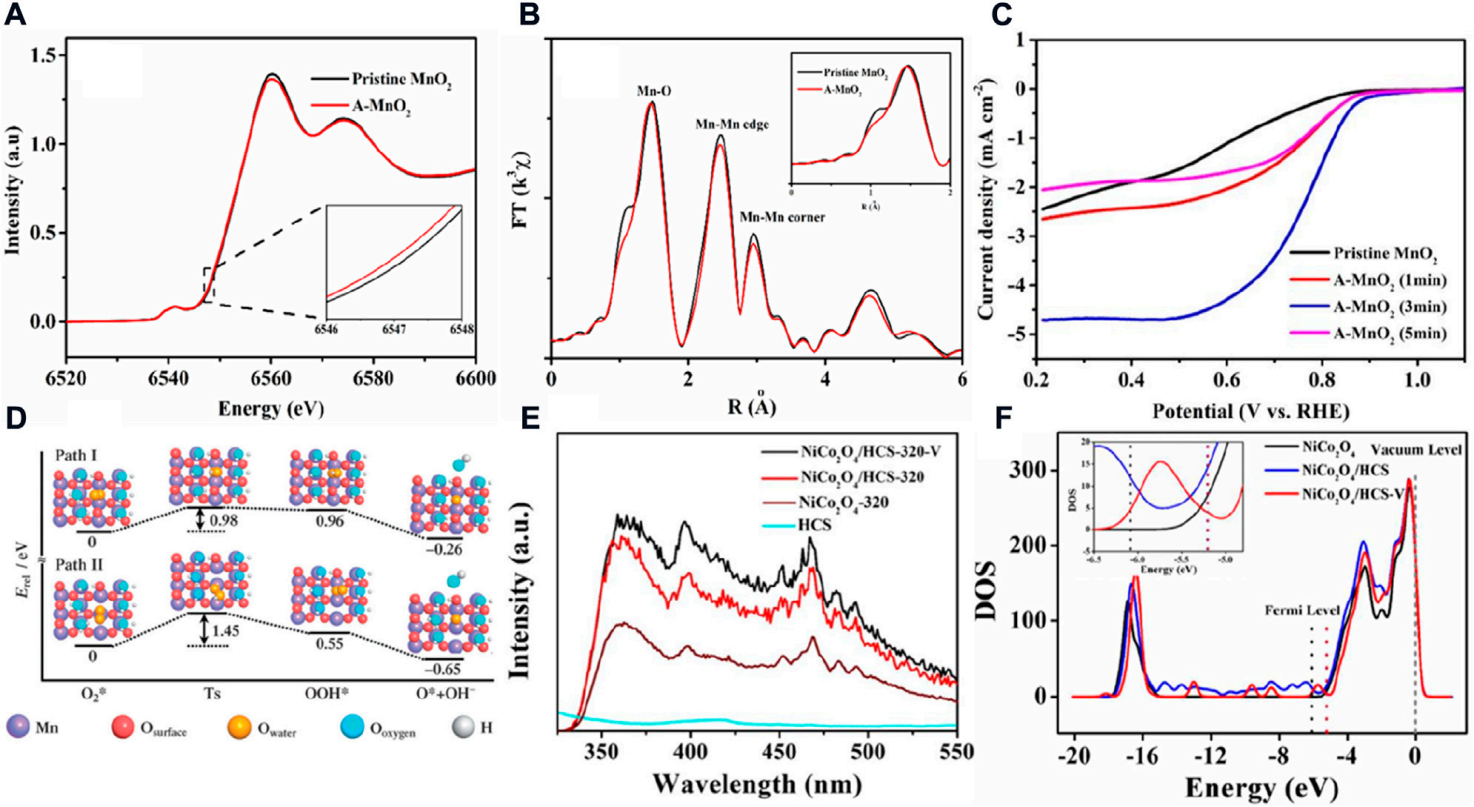
**(A)** Mn K-edge XANES spectra and **(B)** magnitude of k^2^-weighted Fourier transforms of Mn K-edge EXAFS spectra for the treated MnO_2_ and the pristine MnO_2_; **(C)** ORR performance of the treated MnO_2_ with different times and the pristine MnO_2_ (Jiang et al., 2019). **(D)** Energy profiles and configurations of the ORR on MnO_2_ surfaces with one 1) and two 2) oxygen vacancies (Cheng et al., 2013). **(E)** Photoluminescence spectra and **(F)** density of states of the selected catalyst and comparison sample (Yuan et al., 2018).

In addition to the aforementioned catalysts, nano-metal oxides (e.g., Co_3_O_4_, MnO_2_, and Mn_3_O_4_) are also found to exhibit ORR activity in alkaline media (Osgood et al., 2016; Wang et al., 2018). However, the metal oxides are semi-conductor materials with low electrical conductivity in general, which require loading their nano-particles onto the conducting carrier. The different concentrations of oxygen vacancies can be easily prepared on the surface of the metal oxides to improve their intrinsic activity and conductivity by chemical etching; thermal treatment under H_2_, Ar, and N_2_ atmospheres; and plasma treatment. For example, after the Ar-plasma treatment to α-MnO_2_, the energy of Mn K-edge XANES spectra for the treated α-MnO_2_ is 0.2 eV lower than that of pristine α-MnO_2_, and the bond lengths for Mn-O and Mn-Mn are also shorter than those of untreated α-MnO_2_ (Figures 3A,B). These results are sufficient to prove that the oxygen vacancies are constructed by the Ar-plasma treatment. The oxygen vacancies cause severe Jahn–Teller distortion in the MnO_6_ octahedra, resulting in the electronic structure of the surface and making it more suitable for the ORR process. Finally, the α-MnO_2_ treated for 3 min possesses a higher half-wave potential, limiting current, and initial potential than α-MnO_2._ However, no matter whether the plasma treatment time is less or more than 3 min, the ORR activity is greatly reduced, which indicates that the concentration of oxygen vacancy defects has a decisive effect on catalytic activity (Figure 3C). (Jiang et al., 2019) Chen’s group also obtained almost the same conclusion about the O vacancies for enhancing the ORR activity. (Cheng et al., 2013). They prepared MnO_2_ with different concentrations of oxygen vacancies by heat-treating MnO_2_ in Ar or the mixed gas of Ar and H_2_ atmosphere at different temperatures. They further studied the ORR catalytic mechanism of MnO_2_ with oxygen vacancies by DFT calculation. The defect site makes it easier to activate oxygen, and the bond length of the adsorbed oxygen molecules increases significantly on the oxygen vacancy sites. As shown in Figure 3D, the free energy of the rate-determining step of formation OOH^*^ decreases from 3.09 eV on the perfect MnO_2_ (110) surface to 0.96 and 0.55 eV by introducing one and two O vacancies on the MnO_2_ (110), respectively. However, the barrier of the one-oxygen-vacant surface is 0.98 eV, which is lower than the barrier of the two-oxygen-vacant surface. and the one-oxygen-vacant oxide would express a higher ORR catalytic activity for the lower barrier than the two-oxygen-vacant surface. This also reflects well the experimental results, and it is further illustrated that a moderate oxygen vacancy in MnO_2_ is favorable for boosting the ORR performance. Similarly, oxygen defects show a similar mechanism and the relationship of ORR performance in spinel oxides. Mai’s group calcined NiCo_2_O_4_ growing *in situ* on hollow carbon spheres (HCSs) in a low concentration of air atmosphere (molar ratio of N_2_ to air = 1:9). (Yuan et al., 2018). Compared to a perfect crystal formed by calcining in pure air, the higher emission peak at 410 nm of photoluminescence spectroscopy suggests that the calcined catalyst in a low-concentration air atmosphere shows more oxygen vacancies than other selected catalysts (Figure 3E). The oxygen vacancies would change the density of states of NiCo_2_O_4_ and cause the Fermi level/work function to increase/decrease (Figure 3F). The electrons are more easily transferred from the oxygen vacancies to reactants to enhance the ORR activity. It is worth noting that those kinds of metal oxide catalysts are naturally prone to decomposition in acidic media and cannot be used in PEMFCs. Therefore, the application of AFCs is more limited to the development of key materials such as alkaline membranes and non-noble metal catalysts for hydrogen oxidation.

## 3 Summary and outlook

In summary, with the rapid development of solid catalysis theory, nanotechnology, and material characterization technology, more and more attention has been paid to the role of defects in catalysis. Defect engineering shows great promise for the development of practical and affordable electrocatalysts for the ORR. In this review, the relationship between defects and their activity and stability in ORR catalysts was summarized. The defects can effectively accelerate the ORR process by optimizing the local electronic state of the catalyst. The concentration and distribution of structural defects are discussed in detail to deepen the understanding between defects and catalysis. Finally, there are some outlooks for the construction of efficient and low-cost ORR defect catalysts to address the major challenges of clean energy technologies:

1) Precise construction and large-scale synthesis of defect materials for the ORR. The types, contents, and locations of defects greatly affect the catalytic activity. The complexity of defects makes it difficult to carry out qualitative and quantitative controllable construction. To apply the defective catalyst in PEMFCs on a large scale, it is necessary to develop more controllable methods to prepare efficient catalysts by combining defect engineering with other nanotechnology processes (including interface/surface engineering, the control of morphology and size, and regulation of active central composition).
2) *In situ* characterization of the dynamic structural evolution of the defect sites under operating conditions. The high energy of the defect sites is conducive to the adsorption of reaction species, and it is continuously generated and always in dynamic evolution in the catalysis progress. Although some advanced *in situ* techniques can track the structural evolution under ultra-high vacuum, the obtained results may be very different under the restriction condition from the operating conditions. Therefore, to better guide the synthesis of efficient defect catalysts, it is particularly important to track the dynamic evolution and real structure of defects in the catalytic process.
